# Health Economic Outcomes of Subacromial Balloon Spacer Implantation Across Surgical Care Pathways: A Propensity-Matched Analysis of German Statutory Insurance Claims

**DOI:** 10.36469/001c.164920

**Published:** 2026-09-23

**Authors:** Marc Harms, Christoph Pross, Harald Kuhlmann, Tarcyane Barata Garcia, Jan Eßer, Jens Agneskirchner

**Affiliations:** 1 Stryker, Endoscopy Europe, Freiburg, Germany; 2 Stryker GmbH & Co. KG, Duisburg, Germany; 3 Inspiring-Health GmbH, Munich, Germany; 4 WIG2 GmbH — Scientific Institute for Health Economics and Health System Research, Leipzig, Germany; 5 Ostsee Klinik, Bad Schwartau, Germany; 6 Gelenkchirurgie Orthopädie, Hannover, Germany

**Keywords:** subacromial balloon spacer, rotator cuff injuries, propensity score, real-world evidence, claims data, reverse shoulder arthroplasty, healthcare costs

## Abstract

**Background:**

Massive irreparable rotator cuff tears are managed along a surgical spectrum from arthroscopic debridement to reverse shoulder arthroplasty (RSA). The biodegradable subacromial balloon spacer is a minimally invasive option with limited real-world payer-perspective comparative evidence.

**Objectives:**

To quantify postoperative complications, payer reimbursement, and care-pathway utilization for balloon spacer implantation vs four surgical alternatives in routine statutory health insurance (SHI) practice.

**Methods:**

Retrospective propensity-score-matched cohort analysis of anonymized claims from a German SHI database (~4.5 million insured). Adults treated surgically for rotator cuff pathology (ICD-10-GM M75.1) between 2017 and 2020 were included. Balloon spacer patients (n = 99) were matched 1:2 on propensity scores against RSA, open repair, arthroscopic repair, and debridement. Primary outcomes were 12- and 24-month complication rates and index hospitalization costs; secondary outcomes were shoulder-related sick leave and time to first physiotherapy.

**Results:**

Balloon spacer hospitalization costs (€3502) were 65% lower than RSA (€9969) and modestly lower than open repair (€3829); arthroscopic repair (€3245) and debridement (€2464) were less expensive. Twelve-month complications were lower than RSA (1.3% vs 12.2%) and comparable to other comparators. Physiotherapy began earlier after balloon spacer (11.4 days) than RSA (27.6), open repair (20.0), or arthroscopic repair (18.1). In a small exploratory subgroup aged 60 to 64 years (n = 15 to 19 balloon spacer patients per comparison), sick leave duration and indirect costs were directionally lower for the balloon spacer, but these estimates are hypothesis-generating only. The balloon spacer occupied a cost-efficient position in the surgical care pathway, and the cost gap vs RSA was robust in an inpatient-only sensitivity analysis. Residual confounding by indication cannot be excluded.

**Conclusions:**

Within a large German SHI dataset, balloon spacer implantation was associated with substantially lower direct hospitalization costs than RSA, comparable safety to less invasive alternatives, and earlier physiotherapy initiation. A subgroup signal of reduced indirect costs in working-age patients rested on very small samples and should be regarded as hypothesis-generating rather than as input for payer policy modeling. These findings support the device as a de-escalation option for appropriately selected patients in payer systems facing rising arthroplasty utilization.

## BACKGROUND

Surgeons treating massive irreparable rotator cuff tears face a practical problem that clinical trials have not resolved: Which procedure offers the best trade-off between clinical benefit, recovery burden, and cost for the patient in front of them? The answer depends not only on tear morphology but on patient age, functional expectations, comorbidity load, and the constraints of the healthcare system in which the decision is made.^1–3^

Available surgical options form a care pathway of escalating invasiveness, and the four comparators in this analysis span its range. Arthroscopic debridement, often combined with biceps tenotomy or tuberoplasty, removes degenerate tissue and relieves pain without restoring the cuff and is generally associated with a lower rehabilitation burden than reconstructive or arthroplasty procedures.^2,4^ Open and arthroscopic rotator cuff repair reattach the torn tendon with suture anchors and aim at structural restoration, but re-tear rates rise with tear size, age, and fatty infiltration, and both require a period of immobilization before rehabilitation.^5,6^ Reverse shoulder arthroplasty replaces the joint with a semiconstrained prosthesis that recruits the deltoid to restore elevation. It is generally the most resource-intensive option among the procedures considered here and addresses both pain and pseudoparalysis but carries higher complication rates and a prolonged recovery.^1,2,7^ Between these poles, the subacromial balloon spacer competes for adoption as a minimally invasive intermediate option.^8^

The balloon spacer is a biodegradable copolymer balloon implanted arthroscopically into the subacromial space, where it temporarily reduces humeral-acromial contact and restores smoother glenohumeral kinematics without anchors, sutures, or prosthetic components.^9–11^ Over 60 clinical studies have evaluated the device, with systematic reviews and meta-analyses reporting high rates of clinically meaningful improvement.^8,12,13^ At the same time, the START:REACTS trial, a double-blind, multicenter RCT, found no significant benefit over arthroscopic debridement alone, underscoring the importance of patient selection: an irreparable tear with intact subscapularis, intact coracoacromial ligament, absence of advanced glenohumeral arthritis, and a dedicated rehabilitation program appear to be prerequisites for therapeutic success.^14–16^

What remains largely absent from this evidence base is payer-perspective data on how these procedures compare in real-world resource consumption. Economic evaluations have relied on decision-analytic models^17,18^ or single-center time-driven costing studies.^19^ These approaches are valuable but inherently conditional on their built-in assumptions. Real-world claims data from statutory health insurers, capturing actual hospitalization costs, rehabilitation timing, and work absenteeism for unselected patient populations, provide a complementary form of evidence.

This matters because the indications for these procedures overlap far more in daily practice than textbook descriptions suggest. RSA utilization has expanded dramatically over the past decade, with surgeons increasingly applying it to irreparable cuff tears without arthritis,^7,20^ while balloon spacers, partial repairs, and debridement remain viable options for patients who might equally be candidates for arthroplasty. The heterogeneity of surgical selection is not noise to be eliminated; it is the clinical reality that payer-level analyses are uniquely positioned to describe.

Adopting a payer perspective throughout, we used standardized claims data from the German statutory health insurance (SHI) system to conduct a propensity score–matched comparison of the balloon spacer against four alternative surgical strategies: RSA, open repair, arthroscopic repair, and debridement. Endpoints included postoperative complication rates, index hospitalization costs, shoulder-related sick leave, and time to physiotherapy initiation over 24 months.

This study does not compare procedures for identical indications; no claims database permits that level of granularity. Instead, it describes and quantifies the clinical and economic characteristics of treatment pathways as currently deployed in routine care.

## METHODS

### Study Design and Data Source

This retrospective propensity-score-matched cohort study analyzed anonymized claims from the WIG2 research database, which contains longitudinal billing and administrative records for approximately 4.5 million individuals insured under the German SHI system. The database is representative of the German population by age, sex, and morbidity.^21^ The study design follows the STaRT-RWE structured template for planning and reporting real-world evidence studies.^22^ The analysis was conducted from a payer perspective, measuring resource use and care costs as incurred by the statutory insurer. All costs are reported in nominal euros at the year of service under the applicable annual diagnosis-related group (DRG) reimbursement schedule. No inflation adjustment was applied, because the index cost is the contemporaneous DRG reimbursement and matched intervention and control patients are drawn from the same 2017 to 2020 window. Calendar year was not included as a matching covariate. The distribution of index year, compared between matched groups for each comparison, showed no significant imbalance (**Supplementary Table S3**), and the potential for residual temporal confounding is addressed in the **Discussion**.

All analyses were conducted on de-identified SHI claims data in compliance with the General Data Protection Regulation (GDPR) and German social law (§75 SGB X). Because no patient-level identifiers were available and no intervention or patient contact occurred, ethics committee approval and informed consent were not required. The study was not registered in a clinical trial registry, consistent with current conventions for noninterventional analyses of secondary anonymized data.

No published validation study exists for the specific ICD-10-GM (M75.1) or OPS procedure codes used in this analysis within German SHI claims data. As general context for coding integrity, independent Medical Review Board (MDK) audits of German DRG coding reached matching expert conclusions for 73.6% of reviewed coding questions.^23^ The absence of code-specific validation is acknowledged as a limitation in the **Discussion**.

### Study Population

Adults (≥18 years) who underwent surgical treatment for rotator cuff pathology between January 1, 2017, and December 31, 2020, were eligible. Inclusion required an ICD-10-GM diagnosis of rotator cuff lesion (M75.1) documented as a main or secondary diagnosis during the index hospitalization, together with a documented surgical procedure classifiable into one of five mutually exclusive groups:

- Intervention: Balloon spacer implantation (OPS 5-814.c)
- Control 1: Reverse shoulder arthroplasty, RSA (OPS 5-824.21)
- Control 2: Open surgical repair (OPS 5-805.x groupings)
- Control 3: Arthroscopic repair (OPS 5-814.4 ± adjunct codes, eg, 5-814.3)
- Control 4: Debridement (OPS 5-814.3 ± adjunct codes)

Patients were excluded if they had evidence of acute trauma during the index hospitalization (ICD-10-GM S00 to T79), lacked continuous SHI coverage for 24 months before and after the index date, underwent bilateral shoulder surgery at index, had contralateral shoulder surgery during follow-up, or had ambiguous procedural or diagnostic coding that precluded reliable group assignment. Stepwise application of these criteria is summarized in **Supplementary Table S1**.

Death during follow-up was captured through this continuous-enrollment requirement. Because 24 months of uninterrupted post-index SHI coverage was an inclusion criterion, any patient who died within the follow-up window was excluded, as shown in **Supplementary Table S1** (24 months full insurance after index date, deceased excluded). Thus, all analyzed patients had complete 24-month observation. This design avoids unequal follow-up time among included patients, but the findings should be interpreted as conditional on survival and continuous SHI enrollment over the full follow-up period.

### Index Date, Exposure Definition, and Observation Windows

The index date was defined as the date of the qualifying surgical procedure. A 24-month baseline period preceding the index date was used to identify comorbidities, prior healthcare utilization, and medication use. Prior shoulder surgery within this baseline window was not a separate exclusion criterion and was not a covariate in the propensity models, but baseline all-cause costs and utilization were matching covariates, providing partial adjustment for any earlier surgical hospitalization. The frequency of prior ipsilateral shoulder surgery in the matched cohorts is reported in the **Results**. A 24-month follow-up period was used for outcome assessment. Continuous SHI enrollment throughout both periods was required.

Patients were assigned to a single treatment group based on the most specific OPS code documented on the index date. Where multiple qualifying codes were present, an algorithmic hierarchy prioritizing high-specificity codes was applied; cases with irreconcilable coding conflicts were excluded.

Group assignment was fixed at the index procedure and retained for all analyses, an as-started approach analogous to intention-to-treat. A subsequent ipsilateral reoperation or conversion to a different procedure during the 24-month follow-up (eg, a later arthroplasty after a balloon spacer) was permitted and did not change a patient’s group. Such a reoperation was treated as part of the care pathway initiated by the index procedure. It contributes to the complication endpoint when coded as a complication and to follow-up utilization, whereas the primary economic outcome, the index hospitalization cost, is determined at index and is unaffected by any later procedure. Contralateral or bilateral shoulder surgery during follow-up was, in contrast, an exclusion criterion, so that care for the opposite shoulder was not attributed to the index shoulder. The frequency and destination of subsequent ipsilateral procedures in the matched cohorts are reported in the Results and in **Supplementary Table S2**.

### Propensity Score Matching

Propensity score matching (PSM) was performed separately for each intervention-vs-control comparison using 1:2 nearest-neighbor matching without replacement.^24^ A caliper of 0.2 standard deviations of the logit of the propensity score was applied.^25^ Propensity scores were estimated using binary logistic regression. Covariates included age (continuous), sex, the Charlson Comorbidity Index (CCI) score, the 17 individual Charlson comorbidity conditions, and baseline all-cause healthcare costs and utilization documented during the 24-month baseline window. The CCI score and the individual conditions are derived from the same diagnoses and therefore overlap. Because the propensity score functions as a balancing score estimated for prediction rather than as a set of interpretable coefficients, this redundancy may reduce the precision of the individual logistic coefficients, but the score was used for covariate balance rather than coefficient interpretation, and the relevant diagnostic is therefore post-matching balance. Matching was stratified by age subgroup and surgical side. Post-matching standardized mean differences (SMDs) were below 0.1 for the CCI and for every individual condition in all four comparisons, confirming balance on both representations. Statistical comparisons used SMDs with bootstrap 95% confidence intervals throughout, consistent with the paired analytic structure and the descriptive intent of the analysis.

PSM reduces confounding on observed covariates but cannot account for unmeasured factors such as tear morphology, functional status, or surgeon preference. This residual confounding applies equally to all four comparisons and is addressed in the **Discussion**.

### Outcomes

The study defined one clinical and one economic primary outcome. The primary clinical outcome was the postoperative complication rate, identified through predefined ICD-10-GM and OPS codes within two follow-up intervals: day 1 to 12 months (Timepoint 1) and 12 to 24 months (Timepoint 2) after the index procedure. The complete list of complication codes is provided in the **Supplementary Appendix**. These codes capture complications documented in subsequent healthcare encounters; temporal proximity to surgery suggests but does not confirm a causal link to the index procedure.

The primary economic outcome was mean index hospitalization cost, defined as the total DRG-based reimbursement for the hospital episode during which the index procedure was performed, encompassing inpatient stay, surgical procedure, anesthesia, and device-related charges.

Secondary outcomes included shoulder-related sick leave days and reimbursed sick leave costs (derived from ICD-10-GM coded incapacity episodes attributed to M75.1 and related musculoskeletal diagnoses), and time from the index procedure to the first documented physiotherapy prescription. All outcomes were evaluated for the overall matched population and within age-based subgroups (<60, 60-64, 65-69, and ≥70 years).

Two structural features of the German SHI system warrant acknowledgment. First, balloon spacer implantation is reimbursed exclusively in the inpatient setting, whereas arthroscopic repair and debridement are frequently performed on an outpatient basis, so a prespecified sensitivity analysis restricted all comparisons to inpatient-only cases. Second, sick leave episodes are recorded only for employed, statutorily insured individuals who submit a physician-issued incapacity certificate. This introduces measurement asymmetry in indirect cost estimation, though it applies equally across all treatment groups.

Functional outcomes and patient-reported outcome measures were unavailable in the SHI dataset. The selected endpoints are therefore payer-relevant measures of resource use and care trajectory.

### Statistical Analysis

Descriptive statistics were used throughout. Continuous variables are reported as means with standard deviations (SD) and medians with interquartile ranges (IQR); categorical variables as frequencies and percentages. Between-group differences were quantified using standardized mean differences (SMDs) with 95% confidence intervals (CIs). No multiplicity adjustment was applied, consistent with the exploratory nature of the analysis.

Missing index hospitalization cost data were imputed using cohort-specific means. Complete cost data were available for 100% of the intervention group and Control 1 (RSA) among patients with matched controls, 99.0% of Control 2 (open repair), and 99.5% of Controls 3 and 4 (arthroscopic repair and debridement). Given missingness below 2% in all groups, the impact of imputation on results is negligible.

All analyses were performed in R version 4.1 (R Foundation for Statistical Computing, Vienna, Austria), with data preprocessing in SQL. Scripts were version-controlled and independently dual-verified.

## RESULTS

### Study Population

From 28 438 patients who underwent shoulder surgery between 2017 and 2020 in the WIG2 database, sequential application of inclusion and exclusion criteria yielded 12 446 eligible patients: 99 treated with the balloon spacer (<1% of the total cohort), 447 with RSA, 2772 with open repair, 5987 with arthroscopic repair, and 3141 with debridement (**Supplementary Table S1**).

After PSM, the final analytic dataset comprised 99 intervention patients matched 1:2 with 198 controls for the open repair, arthroscopic repair, and debridement comparisons. For the RSA comparison, 21 intervention patients could not be matched within the caliper; the RSA analysis therefore includes 78 intervention patients matched to 156 RSA controls (1:2). This shortfall reflects the clinical distance between balloon spacer and RSA populations: despite a large RSA pool (n = 447), the age- and comorbidity-stratified caliper excluded 21 intervention patients for whom no sufficiently similar RSA control could be identified. As a result, the RSA cost comparison in **Table 3** is based on 78 intervention patients and 156 RSA controls. Post-matching SMDs fell below 0.1 for all covariates, confirming acceptable balance. Baseline characteristics of the matched populations are presented in **Table 1**.

**Table 1.** Baseline Characteristics of the Matched Populations

**Characteristics**	**Intervention (N = 78)**	**RSA (N = 156)**
Age, mean (SD)	69.8 (7.7)	70.1 (8.1)
Sex, female, n (%)	26 (33.3)	50 (32.1)
Location: Left, n (%)	27 (34.6)	58 (37.2)
Subgroup <60 years, n (%)	8 (10.3)	20 (12.8)
Subgroup 60-64 years, n (%)	15 (19.2)	24 (15.4)
Subgroup 65-69 years, n (%)	14 (17.9)	26 (16.7)
Subgroup ≥70 years, n (%)	41 (52.6)	86 (55.1)
Charlson Index, mean (SD)	2.60 (2.70)	2.71 (2.72)

Abbreviations: CCI Charlson Comorbidity Index; RSA, reverse shoulder arthroplasty; SD, standard deviation.

### Prior Surgery and Subsequent Ipsilateral Procedures

In the matched cohorts, prior ipsilateral shoulder surgery during the 24-month baseline was present in 4 of 99 balloon spacer patients (4.0%), in none of the RSA, open repair, and debridement controls (Fisher exact *P* = .022, *P* = .012, and *P* = .012 vs the balloon spacer group), and in 3 of 198 arthroscopic repair controls (1.5%, *P* = .23).

During the 24-month follow-up, at least one subsequent classifiable ipsilateral shoulder operation occurred in 8 of 99 balloon spacer patients (8.1%), 2 of 156 RSA controls (1.3%), 16 of 198 open repair controls (8.1%), 48 of 198 arthroscopic repair controls (24.2%), and 54 of 198 debridement controls (27.3%) (**Supplementary Table S2**). Among controls, most first reoperations repeated the same procedure type (75% to 81%). Among balloon spacer patients, 5 of the 8 reoperations (62.5%) were conversions to RSA.

### Primary Clinical Outcome: Complications

Complication rates in the intervention group were markedly lower than in the RSA group at both follow-up intervals. At 12 months, 1.3% of intervention patients had a documented complication compared with 12.2% in the RSA group (SMD, 0.45; 95% CI: 0.17, 0.72). At 24 months, these rates were 2.6% vs 14.1% (SMD, 0.43; 95% CI: 0.15, 0.70).

Complication rates for the balloon spacer vs open repair (2.0% vs 2.5%; SMD, 0.03), arthroscopic repair (2.0% vs 1.5%; SMD, 0.04), and debridement (2.0% vs 4.0%; SMD, 0.12) were low across all groups, with no statistically significant differences at either timepoint. Full results are reported in **Table 2**.

**Table 2.** Complication Rates (%) Following Surgical Treatment for Rotator Cuff Pathology

	**Y1 Control, n (%)**	**Y1 Intervention, n (%)**	**Y1 SMD**	**Y1 95% CI**	**Y2 Control, n (%)**	**Y2 Intervention, n (%)**	**Y2 SMD**	**Y2 95% CI**
RSA	19 (12.2)	1 (1.3)	**0.45**	**0.17, 0.72**	22 (14.1)	2 (2.6)	**0.43**	**0.15, 0.70**
Open repair	5 (2.5)	2 (2.0)	0.03	−0.21, 0.28	4 (2.0)	2 (2.0)	0.00	−0.24, 0.24
Arthroscopic	3 (1.5)	2 (2.0)	0.04	−0.20, 0.28	2 (1.0)	2 (2.0)	0.08	−0.16, 0.32
Debridement	8 (4.0)	2 (2.0)	0.12	−0.12, 0.36	6 (3.0)	2 (2.0)	0.06	−0.18, 0.31

Abbreviations: CI, confidence interval; RSA, reverse shoulder arthroplasty; SMD, standardized mean differences; Y1, Year 1; Y2, Year 2.

Matched comparisons between intervention (balloon spacer) and each control group. Values shown for Year 1 (day 1 to 12 months) and Year 2 (12 to 24 months) follow-up, SMD, and 95% CI to quantify group differences. Bold indicates 95% CI of SMD, excludes zero.

### Primary Economic Outcome: Index Hospitalization Costs

Mean (SD) index hospitalization costs for the balloon spacer were €3502 (€472) in the RSA comparison and €3496 (€484) in all other comparisons. The cost differences across the four matched comparisons position the balloon spacer in the middle of the procedural cost spectrum (**Table 3**).

**Table 3.** Mean Total Index Hospitalization Costs (€), Matched Comparisons

**Control Group**	**Intervention, n**	**Control, n**	**Mean (SD) Intervention**	**Mean (SD) Control**	**SMD**	**95% CI**
RSA	78^a^	156	3502 (472)	9969 (2145)	**4.2**	**3.7, 4.6**
Open surgical	99	198	3496 (484)	3829 (1184)	**0.37**	**0.13, 0.61**
Arthroscopic	99	198	3496 (484)	3245 (1147)	**−0.29**	**−0.53, −0.04**
Debridement	99	198	3496 (484)	2464 (1622)	**−0.86**	**−1.10, −0.61**

Intervention (balloon spacer) vs each control group following propensity score matching. Values are mean (SD) in Euros. Bold indicates 95% CI of SMD, excludes zero.

Abbreviations: CI, confidence interval; RSA, reverse shoulder arthroplasty; SMD, standardized mean differences; SD, standard deviation.

^a^Intervention n = 78 for the RSA comparison reflects the subset of matched intervention patients with complete DRG reimbursement records; 21 of 99 matched patients lacked index hospitalization cost data and were excluded from this comparison only.

Compared with RSA, balloon spacer costs were 65% lower (RSA mean [SD], €9969 [ €2145]; SMD, 4.2; 95% CI: 3.7, 4.6), the largest absolute difference observed in this study. Compared with open repair (€3829 [€1184]; SMD, 0.37; 95% CI: 0.13, 0.61), the balloon spacer was modestly less expensive.

In contrast, arthroscopic repair (€3245 [€1147]; SMD, −0.29; 95% CI: −0.53, −0.04) and debridement (€2464 [€1622]; SMD, −0.86; 95% CI: −1.10, −0.61) were both significantly less costly than the balloon spacer, as expected for less device-intensive procedures frequently performed in outpatient settings.

### Sensitivity Analysis: Inpatient-Only Costs

Restricting the analysis to inpatient cases only (unmatched) confirmed the direction and magnitude of cost differences. The balloon spacer (€3496 [€484]) remained substantially less expensive than RSA (€10 242 [€4012; SMD, 2.4), open repair (€3985 [€1172]; SMD, 0.55), and, notably, inpatient arthroscopic repair (€3789 [€564]); SMD, 0.56). Debridement remained the least costly procedure at €2942 (€706); SMD, −0.92). Complete results are in **Table 4**.

**Table 4.** Mean Total Index Hospitalization Costs (€), Inpatient-Only, Unmatched

**Control Group**	**Intervention, n**	**Control, n**	**Mean (SD) Intervention**	**Mean (SD) Control**	**SMD**	**95% CI**
RSA	99	447	3496 (484)	10 242 (4012)	2.4	2.1, 2.6
Open surgical	99	2772	3496 (484)	3985 (1172)	0.55	0.35, 0.75
Arthroscopic	99	5987	3496 (484)	3789 (564)	0.56	0.36, 0.76
Debridement	99	3141	3496 (484)	2942 (706)	−0.92	−1.10, −0.71

Abbreviations: CI, confidence interval; RSA, reverse shoulder arthroplasty; SMD, standardized mean differences; SD, standard deviation.

Sensitivity analysis restricted to inpatient cases. Values are mean (SD) in euros. No propensity score matching applied.

Two observations from this sensitivity analysis deserve emphasis. First, the cost advantage of the balloon spacer over arthroscopic repair, absent in the primary matched analysis, emerged when the comparison was restricted to inpatient cases, suggesting that the primary analysis was confounded by the inclusion of lower-cost outpatient arthroscopic procedures. Second, the balloon spacer cost estimate remained virtually unchanged (€3496 in both analyses), confirming that the device is implanted exclusively in the inpatient setting and is unaffected by care-setting selection.

### Exploratory Analysis: Shoulder-Related Sick Leave (Age 60-64 Years)

Shoulder-related sick leave was analyzed across all working-age subgroups. Statistically significant differences in sick leave duration and costs were observed only in the 60-to-64 age subgroup; other age groups showed inconsistent or nonsignificant patterns. Because this subgroup captures individuals nearing retirement age, where recovery duration may disproportionately affect remaining productive years, it is reported in detail. These findings are exploratory, and the sample sizes are small (intervention, n = 15-19 depending on the matched comparison; controls, n = 24-46).

In the RSA comparison (intervention, n = 15; RSA, n = 24), intervention patients averaged 41.4 (87.1) sick leave days vs 113.6 (156.6) days for RSA (SMD, 0.58; 95% CI: −0.07, 1.20). Reimbursed sick leave costs were €759 (SD, €2015) for the intervention vs €5454 (SD, €8245) for RSA (SMD, 0.80; 95% CI: 0.13, 1.50). The days-off difference was directionally large, but the CI included zero; the cost difference was statistically significant.

In comparisons with open repair and arthroscopic repair (intervention, n = 19 in both), sick leave duration was significantly shorter for the balloon spacer: 57.0 (108.9) days vs 132.2 days for open repair (SMD, 0.63; 95% CI: 0.09, 1.20) and 139.4 days for arthroscopic repair (SMD, 0.69; 95% CI: 0.13, 1.20). Corresponding cost differences were also significant: €2013 (€5144) vs €5907 for open repair (SMD, 0.62; 95% CI: 0.07, 1.20) and €7517 for arthroscopic repair (SMD, 0.80; 95% CI: 0.24, 1.40). The comparison with debridement showed a directional advantage for the balloon spacer (57.0 vs 92.6 days; €2013 vs €4626) that did not reach significance (**Table 5**).

**Table 5.** Shoulder-Related Sick Leave (Days and Reimbursed Costs in Euros) at 1-Year Follow-Up, Patients Aged 60 to 64 Years

	**Control, n**	**Interv, n**	**Days, Control (SD)**	**Days, Interv (SD)**	**Days SMD**	**Days 95% CI**	**Cost, Control (SD)**	**Cost, Interv (SD)**	**Cost SMD**	**Cost 95% CI**
RSA	24	15	113.63 (156.62)	41.40 (87.18)	0.58	−0.07, 1.20	5454 (8245)	759 (2015)	**0.80**	**0.13, 1.50**
Open surgical	46	19	132.17 (132.57)	57.00 (108.93)	**0.63**	**0.09, 1.20**	5907 (7479)	2013 (5144)	**0.62**	**0.07, 1.20**
Arthroscopic	41	19	139.41 (134.24)	57.00 (108.93)	**0.69**	**0.13, 1.20**	7517 (8430)	2013 (5144)	**0.80**	**0.24, 1.40**
Debridement	37	19	92.57 (126.66)	57.00 (108.93)	0.31	−0.25, 0.86	4626 (7317)	2013 (5144)	0.42	−0.14, 0.98

Abbreviations: CI, confidence interval; Interv, intervention; RSA, reverse shoulder arthroplasty; SMD, standardized mean differences; SD, standard deviation.

Matched comparisons between intervention (balloon spacer) and each control group. Sick leave duration in days and reimbursed sick-leave costs in euros are reported as mean (SD). Bold indicates 95% CI of SMD, excludes zero.

These results suggest that the balloon spacer may confer meaningful indirect cost savings for working-age patients near retirement, but the small sample sizes preclude confirmatory conclusions. Replication in larger datasets is needed before these findings can inform policy.

### Secondary Outcome: Time to Physiotherapy

Intervention patients initiated physiotherapy earlier than all comparator groups. Mean (SD) time to first physiotherapy prescription was 11.4 (10.1) days for the balloon spacer, compared with 27.6 (32.9) days for RSA, 20.0 (22.2) days for open repair, 18.1 (19.7) days for arthroscopic repair, and 16.9 (19.2) days for debridement (**Table 6**).

**Table 6.** Time (Days) from Index Procedure to Initiation of Physiotherapy

	**Control, Mean (SD)**	**Intervention, Mean (SD)**	**SMD**	**95% CI**
RSA	27.62 (32.86)	11.36 (10.13)	**0.67**	**0.39, 0.95**
Open surgical	19.99 (22.17)	12.71 (17.03)	**0.37**	**0.13, 0.61**
Arthroscopic repair	18.13 (19.72)	12.71 (17.03)	**0.30**	**0.05, 0.54**
Debridement	16.87 (19.15)	12.71 (17.03)	0.23	−0.01, 0.47

Abbreviations: CI, confidence interval; RSA, reverse shoulder arthroplasty; SMD, standardized mean differences; SD, standard deviation.

Matched comparisons between intervention (balloon spacer) and each control group. Values are mean (SD). Bold indicates 95% CI of SMD, excludes zero.

Differences were statistically significant vs RSA (SMD, 0.67; 95% CI: 0.39, 0.95), open repair (SMD, 0.37; 95% CI: 0.13, 0.61), and arthroscopic repair (SMD, 0.30; 95% CI: 0.05, 0.54). The comparison with debridement approached but did not reach significance (SMD, 0.23; 95% CI: −0.01, 0.47).

Earlier physiotherapy initiation is consistent with the minimally invasive character of balloon spacer implantation, which does not require anchors, sutures, or postsurgical immobilization protocols. However, this difference is largely attributable to protocol design rather than patient recovery: RSA and open repair patients are typically immobilized for 4 to 6 weeks before rehabilitation begins, whereas balloon spacer patients face no such restriction. Time to physiotherapy is therefore a process measure that reflects procedural and rehabilitation protocol differences, not a clinical outcome. Whether earlier rehabilitation access translates into faster functional recovery cannot be determined from administrative data.

## DISCUSSION

This propensity-matched analysis describes the economic and clinical profile of balloon spacer implantation relative to four surgical alternatives for rotator cuff pathology. Across all comparisons the balloon spacer showed complication rates comparable to open repair, arthroscopic repair, and debridement, and substantially lower rates than RSA. On cost it occupied the middle of the procedural spectrum, less expensive than RSA and open repair and more expensive than arthroscopic repair and debridement. These patterns held in the inpatient-only sensitivity analysis, where the balloon spacer became cheaper than arthroscopic repair once outpatient cases were removed, confirming that care setting rather than procedural cost drives the primary comparison.

The RSA comparison produced the largest effects in the study. Hospitalization costs were 65% lower, complication rates roughly one-tenth as high, and physiotherapy began 16 days earlier. These differences are clinically plausible, because balloon spacer implantation is a brief arthroscopic procedure with no prosthetic components or obligatory immobilization, whereas RSA is a joint replacement with extended protection protocols. The comparison is also the most vulnerable to confounding by indication. The caliper retained only 156 RSA controls and 78 intervention patients with complete cost data, reflecting the clinical distance between these populations despite observed covariate balance. The 21 unmatched intervention patients were younger and healthier, not older or more comorbid. They averaged 56.6 years against 69.8 in the matched subset, 14 of the 21 were younger than 60 and none were aged 70 or older, and their mean CCI score was 1.05 vs 2.60 (both differences *P* < .001). These patients were not lost from the study, because they remain in the open repair, arthroscopic repair, and debridement comparisons. The RSA cost comparison therefore applies to the older, more comorbid balloon spacer recipients whose profile overlaps with typical arthroplasty candidates, and it excludes the youngest patients for whom reverse arthroplasty would rarely be a realistic alternative. The size of the gap underscores that RSA is a fundamentally more resource-intensive pathway, and the balloon spacer may be read as a de-escalation option for selected patients in whom arthroplasty would be premature. Consistent with this position, 5 of the 8 balloon spacer patients who underwent a subsequent ipsilateral operation within 24 months converted to RSA, whereas reoperations after the comparator procedures predominantly repeated the same procedure type. In these data, the pattern of subsequent procedures is compatible with the balloon spacer being used as an intermediate step in selected patients, with later conversion to RSA remaining possible.

This invites the objection that comparing the balloon spacer with RSA is inappropriate because the two serve different populations. In theory, RSA is indicated for pseudoparalysis or advanced glenohumeral arthritis and the balloon spacer for preserved elevation with an intact subscapularis, a distinction the 2025 American Academy of Orthopaedic Surgeons guideline reinforces.^26^ In practice, the indications overlap. RSA use has expanded to irreparable tears without arthritis, while the balloon spacer is sometimes chosen for patients who might equally receive debridement or partial repair.^7,20^ We do not claim the procedures are interchangeable. We claim that German SHI patients coded with rotator cuff pathology received both, that they can be balanced on observable characteristics, and that the resulting cost and utilization differences are observed, sizeable, and relevant to care-pathway economics, while not constituting causal estimates of comparative effectiveness. The same logic applies to all four comparisons, which should be read as descriptive characterizations rather than causal effect estimates.

These findings extend three prior economic evaluations. Castagna et al modeled the balloon spacer as dominant over RSA in the Italian NHS under an assumption of durable functional improvement that remains contested.^17^ Luthringer et al found reduced operating-room time and personnel cost vs partial repair in a US costing study, although implant cost was higher.^19^ Oeding et al found the balloon spacer optimal in about one-third of probabilistic simulations of a Markov model, particularly under assumptions of lower surgical burden and faster recovery.^18^ We did not model expected costs or utilities. We measured actual DRG reimbursements, rehabilitation timing, and sick leave in unselected patients, providing an empirical anchor consistent with the conditions under which the most recent model favored the device.

The balloon spacer accounted for fewer than 1% of qualifying procedures, which is informative for interpreting both the sample size and the generalizability of the findings. Uptake was low but stable across the study window, at 20 to 28 implantations per year in this database, consistent with an early adoption phase constrained by reimbursement and indication rather than with rapid diffusion. Reimbursement is confined to the inpatient setting and a narrow-coded indication, and the indication is demanding, requiring an irreparable tear with an intact subscapularis and coracoacromial ligament and no advanced glenohumeral arthritis. Ongoing equipoise about the durability of functional benefit has encouraged selective use. The small cohort therefore appears to reflect low real-world uptake during the study period rather than a purely technical sampling issue.

The exploratory sick leave analysis in the 60-to-64 subgroup showed shorter absence and lower reimbursed cost for the balloon spacer across all four comparisons, with nominal statistical significance in selected comparisons. These results rest on 15 to 19 intervention patients per comparison, and the SD exceeded the means, indicating skewed distributions driven by a few prolonged absences. They generate a hypothesis but do not confirm one, and they should not be used for payer policy modeling without larger-scale replication.

Two practical messages follow for systems similar to the German SHI. First, as a de-escalation option, the balloon spacer can substantially reduce direct hospitalization costs relative to RSA while maintaining a complication profile comparable to less invasive procedures, which is material for payers facing expanding RSA utilization.^7,20^ Second, earlier physiotherapy initiation after balloon spacer, although a process measure rather than a clinical outcome, suggests a rehabilitation pathway with fewer structural delays. Whether faster access improves functional recovery is a question for prospective study.

The study has four principal strengths. It used a large, population-representative SHI dataset in which costs reflect actual payer expenditure rather than charge-based proxies. It applied propensity score matching on age, sex, comorbidities, and baseline costs across all four comparisons. It prespecified an inpatient-only sensitivity analysis that confirmed the primary cost findings. And it combined direct costs, process measures, and indirect costs in a single payer-perspective analysis.

Several limitations constrain interpretation. The nonrandomized design cannot exclude residual confounding by indication, because claims data carry no information on tear morphology, functional status, or surgeon preference. This constraint is important for clinical interpretation, but the analysis remains relevant from a payer perspective, because it reflects the type of administrative information routinely available when claims-based reimbursement decisions are made, so the analysis mirrors the informational environment in which coverage and resource allocation are actually decided. Prior ipsilateral shoulder surgery during the baseline was not an exclusion criterion or matching covariate. It was more frequent among balloon spacer patients (4.0%) than in any matched control group (0%-1.5%). This imbalance does not obviously favor the balloon spacer and may indicate a more treatment-refractory intervention cohort, but residual confounding related to prior surgical history cannot be excluded. The primary economic endpoint captures the index hospitalization only, so the reported differences describe the cost of entering each pathway rather than its cumulative 24-month cost, and downstream procedures contribute to the complication and utilization endpoints but not to the cost comparison. The intervention cohort is small at n = 99, which limits power for the subgroup analyses. No functional outcomes or patient-reported outcome measures were available, so all endpoints are administrative proxies and the study complements rather than replaces clinical trials of effectiveness. Complication codes reflect temporal association, not confirmed causality. Sick leave is captured only for employed, statutorily insured individuals. The requirement for complete 24-month follow-up excluded patients who died during follow-up and may therefore limit generalizability to frailer patients, particularly in the more invasive treatment groups. The findings reflect the German SHI reimbursement structure and generalize to other payment models only with caution. No published validation exists for the specific M75.1 or OPS codes used to define the cohort, so cohort assignment relies on the general integrity of DRG coding audited by the MDK rather than on code-specific accuracy.^23^ Finally, calendar year was not a matching covariate. The distribution of index year did not differ significantly between matched groups in any comparison (**Supplementary Table S3**), and the primary cost outcome is the contemporaneous DRG reimbursement under annually fixed national tariffs that changed little over the short study window, so a calendar-time artefact is unlikely, although modest imbalances cannot be excluded in the smaller comparisons.

These results should be read as exploratory real-world evidence that complements, but does not replace, clinical trials. They quantify what treatment pathways cost and how patients move through the health system after surgery. They do not determine which procedure produces the best clinical outcome for a given patient. That question belongs to trials.

## CONCLUSIONS

In this propensity-matched analysis of German statutory insurance claims, subacromial balloon spacer implantation was associated with substantially lower hospitalization costs and complication rates compared with reverse shoulder arthroplasty, comparable safety to open and arthroscopic repair, and earlier physiotherapy initiation across all comparisons. A reduced sick leave burden was observed in a small subgroup of working-age patients near retirement, but this observation is hypothesis-generating and should not be used for payer policy modelling without larger-scale replication. These findings position the balloon spacer as a cost-efficient, minimally invasive option for appropriately selected patients with massive or irreparable rotator cuff tears in payer systems structured similarly to the German SHI. For healthcare systems facing demographic aging, rising arthroplasty utilization, and growing resource constraints, such intermediate options are of increasing strategic relevance. Confirmation in larger datasets and replication in other reimbursement systems are needed before these findings can inform formal coverage policy.

### Disclosures

M.H. is a full-time employee of Stryker Endoscopy Europe. C.P. is a full-time employee of Stryker (Director, Government Affairs and Market Access, Germany/Switzerland/Austria) and participates in the Stryker employee retention stock program. H.K. is Managing Partner/CCO of Inspiring-Health GmbH and reports consulting fees and related travel support from Stryker entities for projects in other business units. T.B.G. is employed by WIG2 GmbH, which received funding from Stryker Germany GmbH for the analysis underlying this manuscript. J.E. reports grants, consulting fees, lecture honoraria, and travel support from Stryker Germany, outside the submitted work. J.A. reports consulting fees and lecture/speaker-bureau honoraria from Stryker Germany GmbH, outside the submitted work.

## Supporting information

Online Supplementary Material
