# Supplementary material for: Health Economic Outcomes of Subacromial Balloon Spacer Implantation Across Surgical Care Pathways: A Propensity-Matched Analysis of German Statutory Insurance Claims
Source: J Health Econ Outcomes Res. 2026 Sep 23;13(2):112–9. doi: 10.36469/001c.164920 (PMC13727280; doi:10.36469/001c.164920)
Supplement: Supplementary file 1 [file jheor_2026_13_2_164920_367868.pdf]

Journal of Health Economics and Outcomes Research

Online Supplementary Material

Health Economic Outcomes of Subacromial Balloon Spacer Implantation Across Surgical Care Pathways: A Propensity-Matched Analysis of German Statutory Insurance Claims. *JHEOR*. 2026;13(2):112-119. doi:10.36469/jheor.2026.164920

Table S1: Stepwise Application of Inclusion and Exclusion Criteria

Table S2: Subsequent Ipsilateral Shoulder Operations Within 24 Months of the Index Procedure (Matched Cohorts)

Table S3: Distribution of Index Year by Treatment Group Before and After Matching

Appendix: Predefined Complication Codes

This supplementary material has been provided by the authors to give readers additional information about their work.

This is an open-access article distributed under the terms of the Creative Commons Attribution 4.0 International License (CCBY-4.0). View this license's legal deed at http://creativecommons.org/licenses/by/4.0 and legal code at http://creativecommons.org/licenses/by/4.0/legalcode for more information.

Table S1. Stepwise Application of Inclusion and Exclusion Criteria

Criteria	Total	Intervention	RSA	Open	Arthroscopic Repair	Debridement
All patients with shoulder joint operation (1st OP) between 2017 and 2020	28 438	119	2419	4033	7985	13 882
Inclusion: Shoulder lesions (M75.1, same case as joint OP)	14 664	112	512	3198	7131	3711
Exclusion: Acute injuries (day of admission, same case)	13 928	112	504	3089	6720	3503
Inclusion: 24 mo full insurance prior to index date	13 743	110	498	3052	6630	3453
Inclusion: 24 mo full insurance after index date (deceased excluded)	13 202	105	470	2928	6382	3317
Exclusion: Bilateral shoulder surgery at index OP; uncoded laterality (n = 6)	13 114	105	470	2911	6334	3294
Exclusion: Contralateral or bilateral shoulder surgery within 24 mo post-index	12 446	99	447	2772	5987	3141

Abbreviations: OP, operation; RSA, reverse shoulder arthroscopy.
Final eligible patient numbers for the intervention group (balloon spacer) and each control group after sequential application of clinical, diagnostic, and insurance-based filters.

Table S2. Subsequent Ipsilateral Shoulder Operations Within 24 Months of the Index Procedure (Matched Cohorts)

A. Frequency of Subsequent Ipsilateral Operations

Index Group	N (Matched)	n with 1 Subsequent Operation	%
Balloon spacer (Intervention)	99	8	8.1
RSA (Control 1)	156	2	1.3
Open repair (Control 2)	198	16	8.1
Arthroscopic repair (Control 3)	198	48	24.2
Debridement (Control 4)	198	54	27.3

B. Destination of the First Subsequent Ipsilateral Procedure (n)

Index Group	Balloon Spacer	RSA	Open Repair	Arthroscopic Repair	Debridement	Total
Balloon spacer (Intervention)	1	5	0	2	0	8
RSA (Control 1)	0	2	0	0	0	2
Open repair (Control 2)	0	2	12	0	2	16
Arthroscopic repair (Control 3)	1	2	1	39	5	48
Debridement (Control 4)	0	3	1	7	43	54

Abbreviation: RSA, reverse shoulder arthroscopy.
Reoperations after open repair, arthroscopic repair, and debridement predominantly repeated the same procedure type (75% to 81% of revised patients). Among balloon spacer patients, 5 of 8 reoperations (62.5%) were conversions to RSA.

Table S3. Distribution of Index Year by Treatment Group Before and After Matching

A. After Matching (Analytic Samples)

Comparison	Group	N	2017	2018	2019	2020	Chi-square *P*
Versus RSA	Balloon spacer	78	18	18	22	20	0.53
	RSA (Control 1)	156	32	49	44	31	
Versus open repair	Balloon spacer	99	20	28	27	24	0.74
	Open repair (Control 2)	198	48	60	51	39	
Versus arthroscopic repair	Balloon spacer	99	20	28	27	24	0.87
	Arthroscopic repair (Control 3)	198	46	48	54	50	
Versus debridement	Balloon spacer	99	20	28	27	24	0.15
	Debridement (Control 4)	198	63	46	40	49	

B. Before Matching (Full Eligible Cohort)

Group	N	2017	2018	2019	2020	Chi-square *P* vs Balloon Spacer
Balloon spacer (Intervention)	99	20	28	27	24	Reference
RSA (Control 1)	447	115	118	122	92	0.65
Open repair (Control 2)	2772	766	715	703	588	0.44
Arthroscopic repair (Control 3)	5987	1538	1421	1499	1529	0.52
Debridement (Control 4)	3141	888	793	763	697	0.37

Abbreviation: RSA, reverse shoulder arthroscopy.
Number of index procedures per calendar year for the balloon spacer group and each control group, before matching (full eligible cohort) and after matching (analytic sample). *P* values are from chi-square tests of independence (year by group) for each pairwise comparison against the balloon spacer group. Statistical power to detect small imbalances is limited in the smaller comparisons, most notably the RSA comparison (78 vs 156 patients).

Appendix. Predefined Complication Codes

ICD-10-GM Code	Description
T81.0	Hemorrhage and hematoma as a complication of surgery, not elsewhere classified
T81.1	Shock during or as a consequence of surgery, not elsewhere classified
T81.2	Accidental puncture or laceration during surgery, not elsewhere classified
T81.4	Infection following surgery, not elsewhere classified
T84.00	Mechanical complication due to joint endoprosthesis: shoulder joint
T84.4	Mechanical complication due to other internally used orthopaedic devices, implants and grafts
T84.5	Infection and inflammatory reaction due to joint endoprosthesis
T84.7	Infection and inflammatory reaction due to other orthopaedic endoprostheses, implants or transplants
T84.8	Other complications due to orthopaedic endoprostheses, implants or transplants
T84.9	Unspecified complication due to orthopaedic endoprosthesis, implant or transplant

Abbreviation: ICD-10-GM, *International Classification of Disease–German Modification.*
Postoperative complications were identified through the following predefined ICD-10-GM diagnosis codes documented in healthcare encounters within the two follow-up intervals (day 1 to 12 months and 12 to 24 months after the index procedure). The code set was applied identically across all five treatment groups.
